# Biliary bypass surgery – Analysis of indications & outcome of different procedures

**Published:** 2013

**Authors:** K.Altaf Hussain Talpur, Arshad Mahmood Malik, Amir Iqbal Memon, Jawed Naeem Qureshi, Ahmed Khan Sangrasi, Abdul Aziz Laghari

**Affiliations:** 1 K.Altaf Hussain Talpur, Liaquat University of Medical & Health Sciences (LUMHS), Jamshoro/Hyderabad, Sindh, Pakistan.; 2 Arshad Mahmood Malik, Liaquat University of Medical & Health Sciences (LUMHS), Jamshoro/Hyderabad, Sindh, Pakistan.; 3 Amir Iqbal Memon, Liaquat University of Medical & Health Sciences (LUMHS), Jamshoro/Hyderabad, Sindh, Pakistan.; 4 Jawed Naeem Qureshi, Liaquat University of Medical & Health Sciences (LUMHS), Jamshoro/Hyderabad, Sindh, Pakistan.; 5 Ahmed Khan Sangrasi, Liaquat University of Medical & Health Sciences (LUMHS), Jamshoro/Hyderabad, Sindh, Pakistan.; 6 Abdul Aziz Laghari, Liaquat University of Medical & Health Sciences (LUMHS), Jamshoro/Hyderabad, Sindh, Pakistan.

**Keywords:** CBD stones, Biliary injuries, Pancreatico-biliary malignancies, Cholecystectomy, Biliary bypass surgery

## Abstract

***Objectives: ***This study reports the indications and outcome of various biliary bypass surgical procedures from a single centre over a period of 10 years.

***Methods: ***This is a prospective observational study conducted over a period of 10 years (January 2001-december 2010). A total of 1500 patients were included, who underwent pancreatico-biliary surgery due to common bile duct (CBD) stones, congenital anomalies of biliary tree, unoperable pancreatico-biliary malignancies, CBD strictures and cases who developed iatrogenic biliary injuries during cholecystectomy (both open & laproscopic) during this period of time. The patients who required biliary bypass surgery were further analysed for indications and outcome.

***Results: ***Out of 1500 patients 83(5.53%) required biliary bypass surgical procedures. The CBD stones were observed as the most common indication (25.3%), followed by CBD injuries after open(10.84%) or laproscopic-cholecystectomy (14.46%), carcinoma head of pancreas (12.05%) and CBD obstruction(14.46%) either due to CBD strictures or unknown distal obstruction. Roux-en-Y-hepatico-jejunostomy (26.51%) was the most frequently performed procedure, followed by choledochoduodenostomy and Roux-en-Y choledocho-jejunostomy (i.e. 25.3% and 12.05% respectively). Roux-en-Y biliary bypass procedure was observed to be associated with better outcome in terms of rate of complications as well duration of hospital stay.

***Conclusion: ***Biliary bypass surgical procedures are the better options to restore the continuity of biliary system in patients with iatrogenic biliary tree injuries and un-operable pancreatico-biliary malignancy. Roux-en-Y biliary bypass procedure is safe and problem solving method in these cases.

## INTRODUCTION

Biliary bypass surgery can be performed to re-route the biliary flow in patients with benign and malignant extrahepatic biliary tract disorders including biliary tree injuries, obstruction and congenital anomalies. The common causes of extrahepatic biliary obstruction are Choledocholithiasis, biliary strictures, sclerosing cholangitis, periampullary growth and carcinoma of head of pancreas.^1^ Obstructive jaundice is the main presenting feature of these disorders and advanced digestive cancer.^2^ Surgical bypass is considered as a treatment of choice for benign biliary diseases whereas a palliation for advanced biliary malignancies.^3^

There are various bilioenteric bypass procedures to deal these problems depending upon the pancreatico biliary pathology. Non-operative techniques are considered as first line of therapy for malignant biliary obstruction.^4^^,^^5^ However in developing countries palliative surgery is the only option available, because sophisticated equipments and expertise are limited to very few centeres.^6^ The different biliary bypass surgical procedures have been advocated but bilio-enteric anastomosis in the form of Roux-en-Y Hepaticojejunostomy or choledochojejunostomy are considered as procedures of choice.^2^^,^^7^^,^^8^ The laparoscopic biliary bypass surgical procedures are also routinely performed in well developed centres but in this study all cases were operated by open surgical methods.

## METHODS

This study was conducted on 1500 patients of either diagnosed or missed primary pancreatico-biliary pathologies or iatrogenic problems encountered during and after conventional open or laparoscopic cholecystectomy. The diagnosed patients with primary biliary disease or secondary to biliary surgery were operated for planned bypass surgical procedures. Those patients of CBD stones having multiple stones, or CBD dilatation >2cm in size or having doubtful distal patency were considered for bypass procedures. However the cases with incidental operative findings of CBD stones, pancreatico-biliary malignancies or biliary tree injuries assessed at the time of surgery or re-exploration after previous surgery were managed according to the merit of problems.

The investigations utilized for diagnosis were liver function test (L.F.T), Ultrasound, CT scan, MRI of abdomen and HIDA scan of hepatobiliary tree. The data regarding number of cases, age, sex and surgical procedures done were collected on specially designed proforma. The main bypass surgical operations performed were choledocho-duodenostomy, Roux-en-Y Choledocho-jejunostomy and Roux-en-Y Hepatico-jejunostomy.

The patients underwent biliary bypass procedures were followed up at one week, then one month after surgery and then every six monthly for a period of two years to assess any long term complication. 

## RESULTS

Out of 1500 cases 83 (5.53%) patients required biliary bypass surgery for different pancreatico-biliary disorders. In these 83 cases 69.87% (N=58) were females and 31.13% (N=25) males with female to male ratio of 2.19:1. Majority of patients presented in 4^th^ & 5^th^ decade (63.85%) with mean age of 47.15 years.

Different pancreatico-biliary problems for which biliary bypass operations done are presented in Table-I. The CBD injuries (36.15%), CBD stones (25.30%) and Ca of head of pancreas (12.05%) were observed as the common indications for these procedures on frequency distribution.

The different bilio-enteric bypass procedures perfomed were Roux-en-Y Hepaticojejunostomy (26.51%), Cholecystectomy and choledocho-duodenostomy (25.30%), Roux-en-Y Choledochojejunostomy (12.05%) as shown in Table-II. The Roux-en-Y Hepatico-jejunostomy was mainly performed in patients having biliary injuries. The overall postoperative morbidity (43.37%) & mortality (2.41%) is given in Table-III whereas hospital stay ranging from one to 4 weeks is given in Table-IV. The most common complications were chest and wound infections, which were seen in cases who were operated for two times and therefore were associated with longer hospital stay. The second most common complication was biliary leak which was observed in majority of cases undergoing choledochojejunostomy, choledochoduodenostomy and cholecystojujenostomy. The bleeding was observed in patients having malignancy. The analysis of the outcome of these procedures showed that the Roux-en-Y biliary bypass procedure was associated with lowest rate of major complications and was considered to be a better option to re-join the biliary duct system to gut.

## DISCUSSION

Disorders of pancreatico-biliary tract affect a significant number of the population all around the world. Majority of the cases are attributed to cholelithiasis and cholestasis due to extra hepatic biliary obstruction.^1^ They can be managed by one of the bilio-enteric bypass procedure depending upon the type of pathology in order to relieve the obstruction or re-communicate the pathway when other alternatives are not feasible. These procedures can be done by conventional open technique or laparoscopically depending upon the facilities available.^9^ In this study only the open surgical procedures were utilized.

**Table-I:** Indications of biliary bypass

*Indication*	*Number of * *Patients *	*Percentage*
Choledochal cyst	4	4.82
CBD* stones + gallstones (Mirizzie synd: 2 cases)	21	25.30
CBD* injuries after open cholecystectomy	9	10.84
CBD* injuries after laparoscopic cholecystectomy	12	14.46
Postoperative CBD* strictures	06	7.23%
CBD* obstruction with unknown aetiology	06	7.23
Carcinoma gallbladder	05	6.02
Common hepatic duct injury	04	4.82
Right Hepatic duct injury	01	1.20
Cholangio carcinoma	03	3.61
Klastiskin tumor	02	2.41
Carcinoma Head of Pancreas	10	12.05

* CBD = Common bile duct

**Table-II:** Biliary bypass procedures

*Name of Procedure*	*Number of* *Patients *	*Percentage*
Cholecystectomy & Choledochoduodenostomy	21	25.30
Choledochoduodenostomy	05	6.02
Choledocho-jejunostomy	05	6.02
Choledochojejunostomy+entero-enterostomy	05	6.02
Roux-en-Y Choledochojejunostomy	10	12.05
Cholecystojejunostomy	3	3.61
Cholecystojejunostomy + Entero Enterostomy	04	4.82
Roux-en-Y Cholecystojejunostomy	01	1.20
Hepaticojejunostomy+ Entero-enterostomy	05	6.02
Hepatico-Jejunostomy (Roux-en-Y)	22	26.51
Triple bypass	02	2.41

**Table-III:** Complications of surgery

*Complication*	*Number of * *Patients *	*Percentage*
Bleeding	05	6.02
Chest infections	09	10.84
Wound infection	08	9.64
Sub-phrenic collection	03	3.61
Sub hepatic collection	02	2.41
Postoperative adhesions	02	2.41
Biliary leak	07	8.43
Death	02	2.41

Postoperative Mortality 2.41%, Morbidity = 43.37%

**Table-IV:** Hospital stay

*Hospital stay *	*Number of * *Patients*	*Percentage*
One week	28	33.73
Two weeks	25	30.12
Three weeks	21	25.30
Four weeks	9	10.84

The decision of bilio-enteric bypass procedures in all these patients was based on the type of pathology and most of these patients had at least one strong indication for such procedure. Choledocho-duodenostomy was used either as single procedure (6.02%) for biliary strictures or as a combined procedure (25.30%) for cholelithiasis and choledocholithiasis including two cases of type II Mirrizie’s syndrome. However Moumen M et al has used bilio-intestinal anastomosis in 20% of cases for common bile duct stones.^10^ Overall this procedure provides effective relief of obstructive jaundice in benign biliary tract conditions but it is not universally used for malignant biliary obstruction.^11^^,^^12^ The treatment of type II Mirizzie’s syndrome described by Chan CY et al^13^ is cholecystectomy & hepaticojejunostomy which is different from this study.

Choledochojejunostomy is preferred method for bilio-pancreatic malignancy^8^^,^^13^ because majority of cases are not curable & present with obstructive jaundice^6^ thus most commonly performed method is cholecystojejunostomy for irresectable pancreatic carcinoma^14^ along with routine gastrojejunostomy.^15^ In this study different variants of choledochojejunostomy in the form of side to side choledochojejunostomy (6.02%), side to side choledochojejunostomy with enter-enterostomy (6.02%) and Roux-en-Y choledochojejunostomy (12.05%) were performed mainly for benign conditions such as biliary strictures, biliary obstruction of unknown aetiology & biliary injuries. However in case of advanced pancreatic cancer simple cholecysto-jejunostomy (3.61%), cholecystojejunostomy with entero-enterostomy (4.82%) and Roux-en-Y choleycstojejunostomy (1.20%) were performed along with triple bypass in 2.41% of cases. The most commonly preformed procedure by Khan IM et al^16^ was also triple bypass. However palliation of jaundice with unresectable pancreatic cancer can be achieved by endoscopic transpapillary biliary stenting, percutaneous transhepatic biliary stenting^8^^,^^17^^,^^18^ photodynamic therapy and radio-chemotherapy. Biliary bypass operation confers better survival as compared to metallic stents in the treatment of unoperable distal malignant biliary obstruction.

Major indications for hepatico-jejunostomy are benign or iatrogenic strictures and injuries of biliary system.^19^^,^^20^ The appropriate treatment of major bile duct injuries is mandatory in order to avoid serious complications and bile reconstruction is best carried out by Roux-en-Y Hepatico-jejunostomy.^21^ In this study simple hepatico-jejunostomy with entero-enterostomy (6.02%) and Roux-en-Y Hepaticojejunostomy (26.51%) was carried out in biliary injuries & strictures above the level of cystic duct & Klatiskin tumor. However Bakhsh R et al used Roux-en-Y Hepaticojejunostomy in 40% of cases^22^ with fibrosed CBD which is quite high from this study.

The morbidity and mortality of these procedures is relatively high than simple routine operations because they are time consuming, sophisticated and complicated procedures. However this study still shows low morbidity (43.37%) and mortality (2.41%) as compared to Hussain Z et al^1^ study which shows higher morbidity of 52%.

## CONCLUSION

Results of the study suggested that the Roux-en-Y biliary bypass is the safe and problem solving procedure for major bile duct injuries showing better outcome in terms to re-route the biliary flow and to show long term benefit to the patients as compared to other procedures done for the same reason. However further prospective studies are required to confirm these findings on a large sample size.

## Authors contribution

1. K. Altaf Hussain Talpur: Manuscript writing.
2. Arshad Mahmood Malik: Collection of ten years data.
3. Amir Iqbal Memon: Data collection and statistical analysis.
4. Javed Naeem Qureshi: Editing of manuscript.
5. Ahmed Khan Sangrasi: Literature search.
6. Abdul Aziz Laghari: Critical review and final approval of the manuscript.
